# Equivalence of information production and generalised entropies in complex processes

**DOI:** 10.1371/journal.pone.0290695

**Published:** 2023-09-06

**Authors:** Rudolf Hanel, Stefan Thurner

**Affiliations:** 1 Section for Science of Complex Systems, CeMDS, Medical University of Vienna, Vienna, Austria; 2 Complexity Science Hub Vienna, Vienna, Austria; 3 Santa Fe Institute, NM, Santa Fe, NM, United States of America; University of Porto Faculty of Engineering: Universidade do Porto Faculdade de Engenharia, PORTUGAL

## Abstract

Complex systems with strong correlations and fat-tailed distribution functions have been argued to be incompatible with the Boltzmann-Gibbs entropy framework and alternatives, so-called generalised entropies, were proposed and studied. Here we show, that this perceived incompatibility is actually a misconception. For a broad class of processes, Boltzmann entropy –the log multiplicity– remains the valid entropy concept. However, for non-i.i.d. processes, Boltzmann entropy is not of Shannon form, −*k*∑_*i*_*p*_*i*_ log *p*_*i*_, but takes the shape of generalised entropies. We derive this result for all processes that can be asymptotically mapped to *adjoint* representations reversibly where processes are i.i.d. In these representations the information production is given by the Shannon entropy. Over the original sampling space this yields functionals identical to generalised entropies. The problem of constructing adequate context-sensitive entropy functionals therefore can be translated into the much simpler problem of finding adjoint representations. The method provides a comprehensive framework for a statistical physics of strongly correlated systems and complex processes.

## Introduction

To know the information content of a process, a system, a source, a signal, or a sequence, one uses entropy to quantify it. If systems or processes are independent identically distributed (i.i.d.), ergodic and stationary in their probabilities, it is known what to do: one uses the expression [1],

$$\begin{array}{c} H\left(p\right)=-\sum_{i=1}^{W}\;p_{i}\;\text{log}\;p_{i}\;, \end{array}$$ (1)

where *i* = 1, 2, …, *W* are the states the system can take and *p*_*i*_ is the probability to observe them. So-called Shannon entropy is given by *S*(*p*) = *kH*(*p*), where *k* > 0 is a constant that specifies the units of entropy, in statistical physics *k* = *k*_*B*_ is the Boltzmann constant; if we measure information in bits per symbol, *k* = 1/log 2. If the system or process of interest is not i.i.d., ergodic, or in stationary equilibrium, then it becomes less clear what to do in order to obtain its correct information content. In principle there are two conceptually very different paths to solve the problem.

**The first way** (which we present here in this paper) is to look at the *information production* of the process or system. Consider a process, *X*, that emits signals *x*(*T*) of length *T* with *x*(*T*) = *x*_*T*_*x*_*T*−1_…*x*_1_. Such an ordered list/sequence of elements *x*(*T*) is often referred to as a *T*-tuple (or *T*-gram). Every symbol, *x*_*t*_, in the signal is an element of a fixed sample space or an alphabet, A, that contains all possible states *x*_*t*_ can take. For example, if you think of *X* as a text-producing author, states are the letters from the English alphabet, $A^{\text{letters}}$, or in a “binary alphabet”, $A^{0,1}$ of zeros and ones, once the text is stored on a computer. In non-i.i.d. systems, symbols within sequences will in general be correlated in one way or another. Those correlations –that may extend over many different scales in the system– carry information about the system. Using the marginal distribution functions of the occurrences of states (letters) in Eq (1) will then certainly not provide the correct information content of the process. However, if we know the probability distribution, *p*_*T*_, to observe entire sequences, *x*(*T*), we compute the information production of process, *X*, as

$$\begin{array}{c} I\left(X\right)=\underset{T\to \infty }{\text{lim}}\frac{1}{T}kH\left(p_{T}\right)\;, \end{array}$$ (2)

see also Text 2 in S1 File for details. This changes the perspective from individual symbols, events, or states to entire sequences, or paths. From an information theoretic point of view, *I*(*X*) measures the average number of bits required to reversibly encode samples of *X* into bit-streams that can be sent through information channels and measure information production in *bits per emitted symbol*, i.e., *k* = 1/log(2). Note that *kH*(*p*_*T*_) measures bits per *T*-tuple, i.e. per path-segment-of-length-*T*. Therefore, by using *k*_*T*_ = *k*/*T*, one measures again bits per emitted symbol of the original alphabet. However, the number of all possible *T*-tuples –the size of the new “alphabet”– is enormous if *T* is large. If the sample space contains A=|A| symbols, the size of the alphabet for all paths is *A*^*T*^. The sum over all states and knowing their probabilities, *p*_*T*_, will in general be impossible.

The reason why *I*(*X*) is the true information production rate of process *X*, is because there exist representations of *X* in terms of other processes, *Y*, such that sequences, *x*, presented in the symbols from the initial alphabet, $A_{0}$, can be rewritten into sequences, *y*, using other symbols from a much larger alphabet. The definition of information production, *I*(*X*), asymptotically uses the largest alphabet containing each possible sequence as a unique symbol, which –as a consequence– are statistically independent. In other words, all structure (correlations) gets absorbed into new symbols belonging to an *extended alphabet*. Only, the definition of information production uses the largest possible alphabet; the alphabet of *T*-tuples over the original alphabet and *T* → ∞.

However, typically, there exist much smaller alphabets that can capture all structures of a process or system. We say *Y* is *adjoined* to *X* (see subsection *Constructing adjoint process spaces* for details) if *Y*s symbols are uncorrelated and, thus, *H* measures the correct information content. In general, the symbols, *z*, in the extended alphabet, encode for a number, *ℓ*, symbols in the original alphabet. For instance, if *z* is a symbol in the alphabet of *T*-tuples, then *T* = *ℓ*(*z*), *z* consisting exactly of *T* letters of the original alphabet. The average length, $\bar{\ell }=\langle \ell \rangle =\sum_{z}f_{z}\ell \left(z\right)$, of original symbols emitted per symbol in the extended alphabet increases with the size of the extended alphabet. *f*_*z*_ = *p*_*z*_(*Y*) is the distribution function of letters *z*. Consequently, the unit of information, *k*, *adapts* to the “complexity” *ℓ*(*z*) and $k\to k/\bar{\ell }$.

For an example of how one can encode information about correlations of a process *X* on all relevant scales imagine an initial alphabet of Latin letters and extend it to a series of extended alphabets: one that contains syllables in addition to letters, one that adds word-fragments, one that includes words, one with frequent word combinations, one with phrases, and so on. This sequence of alphabets is nested in the sense that they contain each other, $A_{0}\subset A_{1}\subset A_{2}\subset \cdots \subset A_{n}\cdots$. With any one of these alphabets, say $A_{n}$, one can sample “text” from it by using the associated marginal distributions, *p*_λ_(*n*), of its elements, λ. With increasing *n*, the resulting artificial text samples will more and more resemble the English text body from which the marginal distributions *p*_λ_(*n*) were derived; see Text 1 in S1 File for the examples given by C. Shannon. We can find particular sequences of alphabets such that each alphabet, $A_{n+1}$, contains exactly one more symbol than $A_{n}$, by applying reversible substitutions of symbols, which we refer to as “parsing rules”. For details, see Text 3 in S1 File.

**The second way** is to directly capture the correlations and structures in non-i.i.d. systems / processes in a generalised functional form of the entropy, which –typically– looks more complicated than Eq (1), an approach that so far has been successful for a handful of processes. Generalised entropy functionals are usually expressed in terms of marginal distributions in the “original alphabet”. These *generalised entropies* have been extensively studied for several decades [2–6] from different angles, generally for systems with strong or long-range correlations [7–9], that are non-ergodic, internally constrained [10–12], or for systems out of equilibrium [13–15].

For non-i.i.d. systems or processes it is essential to specify the context in which the term entropy is used, whether one talks about information theory, thermodynamics, or the maximum entropy principle (MEP) [16]. While thermodynamic aspects of such systems, especially the existence of well defined thermodynamic potentials (and as a consequence, temperature) is heavily debated, there is wide consensus that *entropy production* (the physical analogue of information production) remains a valid concept, also for these systems. For thermodynamic considerations away from equilibrium and i.i.d. and applications e.g. to neural correlations see for instance [17–19].

Here we will not focus on thermodynamic aspects of entropy, but on the original context envisioned by Boltzmann: its power to predict a particular macro state from knowing the number of micro states (multiplicity) corresponding to it. This allows one to predict typical distribution functions and derive functional relations between macro state variables (expectation values of respective observables). This view that is tightly related to the MEP is not restricted to physics and is not limited to i.i.d. processes. For specific cases the respective MEP functionals, the generalised entropy and cross entropy, have been explicitly constructed [5, 6]. In particular, if multiplicity and probabilities are multiplicatively separable in the assymptotic limit, [5], a clear definition of cross entropy is possible also for non-i.i.d. systems.

While the Boltzmann entropy concept remains untouched (log multiplicity) its functional form, i.e., the generalised entropy functional, depends on the context of the process, *X*, and the process class, Φ, to which it belongs to. Some examples for different process classes include i.i.d. processes, exchangeable and polynomial mixture processes [20], Polya processes [21], sample space reducing processes [6], and processes describing structure forming systems [22].

The idea behind generalised entropies is to quantify entropy as the logarithm of the number of micro states (multiplicity) of process, *X*. It is based on the marginal distribution function, *g*_*i*_ = *p*_*i*_(*X*), of symbols *i* from a sample space (here the original alphabet is $A_{0}$) that compose a functional, $\tilde{S}\left(g\right)$, such that it captures the structural information in *X*. Similarly, one obtains generalised expressions for cross entropy and information divergence. Those functionals effectively capture arbitrarily complicated relations between symbols of the sample space (original alphabet) in terms of marginal symbol frequencies, *g*, of the process *X*. Generalised entropies (that fulfil the first Shannon-Khinchin axiom) do not explicitly depend on system parameters that identify a process within a process class or other details. It is obvious that in general, constructing such a functional may be complicated and has not been achieved convincingly, except for a few exceptions, e.g. [6, 21]. As we will see, one can reconstruct (or at least approximate) such functionals from data—at least in principle, since there exist fundamental limits to reconstructing generative grammars from data on the basis of statistical inference alone, [23, 24], a fact also captured in Chaitin’s incompleteness theorem, [25]. In other words, the question of whether some data, a particular sequence, *x*, contains regular structures that can be used to compress it, may become undecidable.

**Fig 1:**
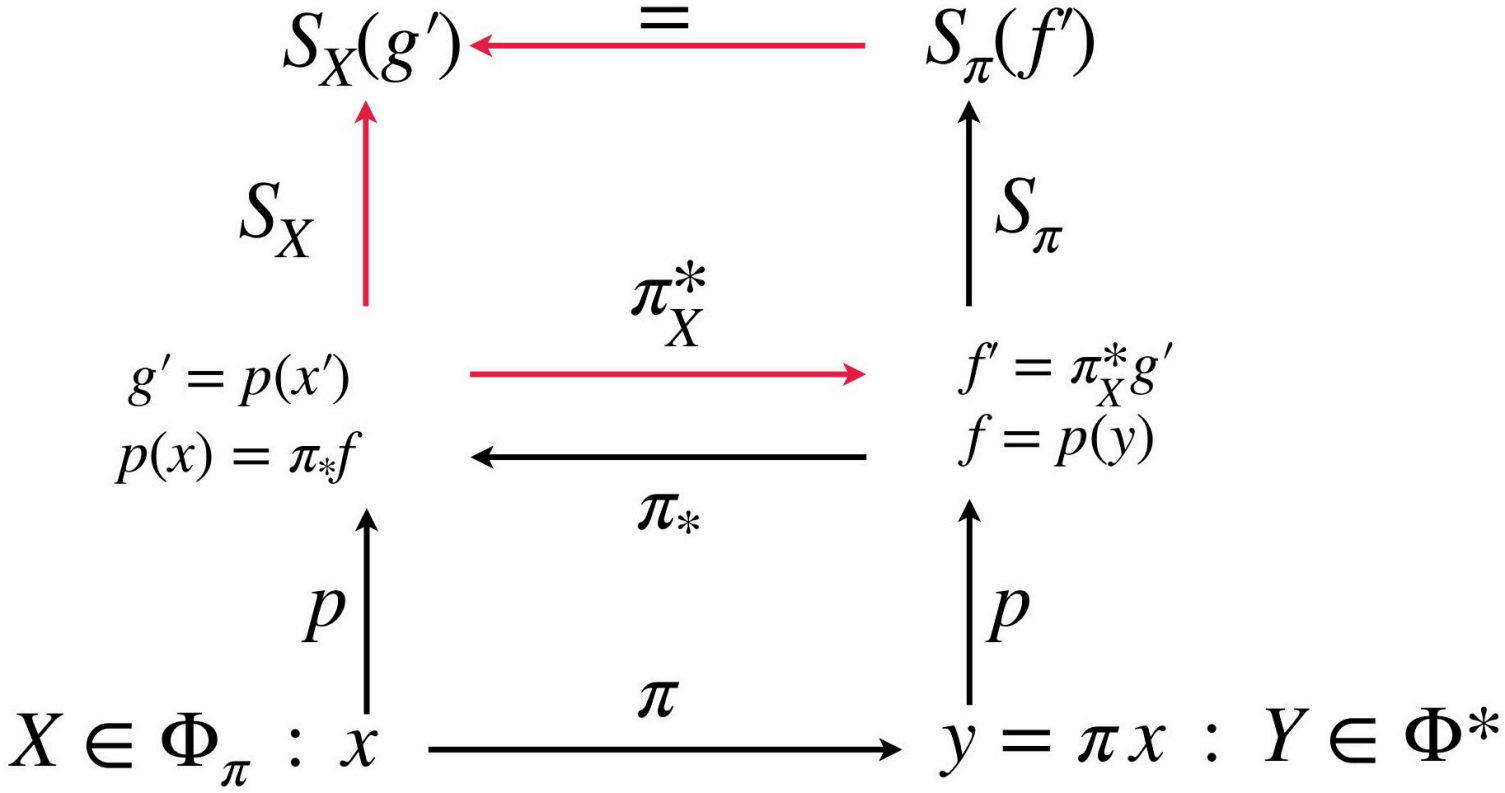
Diagram of the relations between of distribution functions and entropies over the sample space and adjoint samples space. We consider a process, *X* over alphabet A with adjoint process *Y* = *πX* over the adjoint alphabet $A^{*}$. *Y* is i.i.d. and therefore fully characterized by the marginal distribution of letters it samples from, i.e. asymptotically data *y* = *πx* is fully characterized by the relative frequency distribution function *f* = *p*(*y*). Φ* is the set of all i.i.d. processes over $A^{*}$. Therefore Φ_*π*_ = *π*^−1^Φ* is the class of processes that naturally generalize *X* ∈ Φ_*π*_. *f* can be projected to the marginal distribution function *g* = *p*(*x*) = π_*_p(*y*) = π_*_f. Conversely, for a particular process, *X*, we can lift the distribution function *g* to the associated adjoint distribution and get $\pi_{X}^{*}g=\pi_{X}^{*}p\left(x\right)=p\left(y\right)=f$. Since *Y* is i.i.d. over the adjoint sample space one can measure information production simply by using Shannon entropy with the adequate Boltzmann factor *k*_*Y*_ (adapted to the distribution function *f*). The commutative diagram therefore defines the process specific generalised entropy *S*_*X*_ over the sample space of the process class Φ_*π*_ = *π*^−1^Φ*.

Here we show that the two approaches, information production and the generalised entropy functionals can be mapped to one another, meaning that they are the same. The diagram in Fig 1 schematically shows the basic idea: We first use the method of parsing rules to construct an adjoint representation, *Y*, of a given process *X*, and write *Y* = *πX*. Here *π* is a map that reversibly encodes all structures in *X*, such that process *Y* in its new extended alphabet is i.i.d. and is therefore fully described by their marginal distribution function, *f*_*z*_ = *p*_*z*_(*Y*), where *z* is again a letter from the extended alphabet. Consequently, the Shannon information measure with the appropriate unit of information is adequate (first way). Next, we project the marginal distributions from the adjoint representation, *f*_*z*_, to the original alphabet and, in a last step, we identify the process specific “pull-back” information measures, *S*_*X*_, $S_{X}^{\text{cross}}$, and *D*_*X*_, which get precisely defined in Eq (14) below, that takes distribution functions over the original alphabet, with the corresponding generalised information measure (second way) by adequately lifting distribution functions over the original alphabet to distributions over the adjoint alphabet.

For the proof we use the *minimal description length* (MDL) (see also Text 2 in S1 File), the length of the shortest encoding that fully represents the data. In this context, we shall see that information production is tightly related to the notion of Kolmogorov complexity [25–27]; For a brief discussion, see Text 4 in S1 File. We explicitly demonstrate the method in an example for the class of sample space reducing (SSR) processes [21] that provide simple, analytically tracktable models for driven dissipative systems, that typically exhibit distribution functions that are power laws. Their generalised entropy is exactly known for arbitrary driving rates [6, 21].

The purpose of the paper is to show that indeed, *S*_*X*_(*X*) = *k*_*Y*_*H*(*Y*), represents generalized entropies in the second way. The proof is given constructively in the following section.

## Results

The key tool used in the following are simple substitution rules, *parsing rules*, that allow us to reversibly re-code (possibly correlated) data streams into new symbol streams that no-longer carry structure; see Text 3 in S1 File. The structure of a parsing rule we refer to as a *template*. A particular substitution rule derived from a template we refer to as a *parsing rule*.

The simplest parsing rule template, that we refer to as the *elementary* template, can be denoted by [*r s* → *m*], meaning that two symbols *r* and *s* that appear together are substituted with a new symbol *m*. In the following we will associate *m* also with the symbol index, i.e. it is the *m*-th symbol in an alphabet.

To recode data one may choose a suitable set of parsing rule templates. We speak of a *relevant* set of parsing rule templates if (i) one can extract the full information content of a process, *X*, asymptotically, solely by using parsing rules from the set of templates, and (ii) if omitting one template from the set does not allow one to do so. In the following we focus on processes for which the elementary parsing rule template forms a relevant set. However, the arguments presented here extend naturally to more general sets of parsing rule templates; see Text 3 in S1 File.

A generalization of the framework we present from categorial to continuous random variables can be considered via first coarse-graining the continuous dynamics and then taking the limit to ever finer scales of coarse graining, however at considerable measure theoretic cost. Genuinely reformulating the theory in a framework of path-integrals will mainly be limited by translating the concepts of alphabet and parsing rules, which are inherently discrete objects, into a framework of continuous variables.

### Constructing adjoint process spaces

Decidability issues may forever limit our ability to design algorithms for reconstructing optimal adjoint representations from data that are not context specific. On the other hand, for any finite data over finite sample spaces and finite relevant set of parsing rule templates one can in principle find the optimal proxy to an adjoint representation by extensive search algorithms, even though time issues forbid such extensive searches in practical implementations. The general procedure underlying the construction of adjoint representations always remains the same.

Suppose *X* is a process that emits symbols *i* drawn from the alphabet $A_{0}=\left\{1,2,\cdots ,W\right\}$, where *W* is the number of symbols. *X* generates data streams, *x*(*t*) = *x*_*t*_*x*_*t*−1_⋯*x*_1_, where every *x*_*t*_ is one of the available symbols in $A_{0}$, which contains *W* elements. Consider now two letters *r*_1_ and *s*_1_ such that the pair *r*_1_*s*_1_ ≡ *x*_*τ*_*x*_*τ*−1_ for some positions *τ* in the data *x*, has been identified to contain relevant information (e.g. because the pair is over-expressed), then we can rewrite the pair *r*_1_*s*_1_ by a new letter *m*_1_ = *W* + 1, which will become the first letter extending alphabet $A_{0}$ to $A_{1}=A_{0}\cup \left\{W+1\right\}$, with the parsing rule *π*_1_ = [*r*_1_ *s*_1_ → *W* + 1].

We can iterate and produce parsing rules *π*_*n*_ = [*r*_*n*_ *s*_*n*_ → *W* + *n*], with letter indices *r*_*n*_ < *W* + *n* and *s*_*n*_ < *W* + *n*. Where *π*_*n*_ maps data over the alphabet $A_{n-1}$ to data over $A_{n}=A_{n-1}\cup \left\{W+n\right\}$. Note that the parsing rules *π*_*n*_ can be uniquely inverted, i.e. we can expand data over $A_{n}$ to data over $A_{n-1}$ using the inverse map $\pi_{n}^{-1}=\left[W+n\;\to \;r_{n}\;s_{n}\right]$. In other words the inverse parsing rules can be thought of being part of a “generative grammar”, [23]. We therefore can construct a sequence of maps *π*(*n*) = *π*_*n*_*π*_*n*−1_⋯*π*_1_ such that data *x* can be mapped to representations *y*_*n*_ = *π*(*n*)*x*. At every *parsing level*, *n*, we get a corresponding distribution function of the re-coded data, *p*_*z*_(*y*_*n*_), with a letter index *z* = 1, 2, ⋯, *W* + *n*.

The Kraft and McMillan theorems [28, 29] tells us that if all that we know about a process are its marginal relative frequencies, *g*_*i*_, at which symbols *i* occur, there exists a shortest reversibly encoding of the data, *x*, of characteristic length, $L^{\text{min}}$, the *minimal description length* (MDL) that gives the theoretically achievable minimal length of *x* (in units of bits). $L_{\text{min}}$ is a lower bound for the true MDL, *L*(*x*), that can only be attained asymptotically. The theorems state that

$$\begin{array}{c} kH\left(g\right)=\underset{t\to \infty }{\text{lim}}\;\frac{1}{t}L\left(x\left(t\right)\right)\;, \end{array}$$ (3)

with *k* = 1/log(*b*), where *b* is the basis in which information is measured. For bits we typically have *b* = 2. *kH*(*g*) is the MDL in bits per symbol and $L^{\text{min}}\;=\;tkH\left(g\right)$ is the minimal number of bits required to encode messages of length *t*.

For data *x*(*t*) of length *t* we find a sequence of representations *y*_*n*_(*t*) = *π*(*n*)*x*(*t*). Suppose now that for every *t* we find a parsing level n*(*t*) such that *y*_n*(*t*)_(*t*) is a representation of the data *x*(*t*) that cannot be distinguished from an i.i.d. process (which we indicate here by a *). It follows that *y*_n*(*t*)_(*t*) is entirely determined by its marginal distribution of letters *p*_*z*_(*y*_n*(*t*)_(*t*)) and obtain asymptotically $kH\left(p\left(y_{n^{*}\left(t\right)}\left(t\right)\right)\right)≃\frac{1}{|y_{n^{*}\left(t\right)}\left(t\right)|}L^{\text{min}}\left(y_{n^{*}\left(t\right)}\left(t\right)\right)$, where ≃ means asymptotically identical. With |.| we indicate the length of the sequence of letters in numbers of letters of the underlying alphabet. For instance we have |*x*(*t*)| = *t* and |*y*_*n*+1_(*t*)| ≤ |*y*_*n*_(*t*)|. Then, we can asymptotically measure the information production of the process *X* to be

$$\begin{array}{c} \frac{1}{t}L^{\text{min}}\left(x\left(t\right)\right)≃\frac{|y_{n^{*}\left(t\right)}\left(t\right)|}{\left|x(t)\right|}kH\left(p\left(y_{n^{*}\left(t\right)}\left(t\right)\right)\right)\;. \end{array}$$ (4)

As discussed above, the “complexity” of a symbol *ℓ*(*z*) is the number of letters in the original alphabet it codes for. As a consequence we can compute the average symbol complexity for data *y*_n*(*t*)_(*t*) to be given by ${\langle \ell \rangle }_{Y_{n^{*}\left(t\right)}}≃\left|x\left(t\right)|/\right|y_{n^{*}\left(t\right)}\left(t\right)|$. As a consequence, we get for the adjoint process *Y* = lim_*t* → ∞_*Y*_n*(*t*)_ that ${\langle \ell \rangle }_{Y}={\text{lim}}_{t\to \infty }{\langle \ell \rangle }_{Y_{n^{*}\left(t\right)}}$. The adequate unit of information, *k*_*Y*_, for measuring information production, *k*_*Y*_*H*(*p*(*Y*)), therefore is given by

$$\begin{array}{c} k_{Y}\left(x\right)=\frac{k}{{\left\langle \ell \right\rangle }_{Y}}\;. \end{array}$$ (5)

For simplicity, suppose there is a maximal n* that holds for all *t*. Since we assumed that *y*_n*_ is already indistinguishable from an i.i.d. process, applying another parsing rule would only compress the data without changing its MDL. This means, $k_{Y_{n*+1}}H\left(p\left(Y_{n^{*}+1}\right)\right)=k_{Y_{n^{*}}}H\left(p\left(Y_{n^{*}}\right)\right)$. If, conversely, we look at a parsing level, *n*, where the adjoint process is not yet i.i.d., then we can find a parsing rule, *π*_*n*+1_, such that $k_{Y_{n+1}}H\left(p\left(Y_{n+1}\right)\right)<k_{Y_{n}}H\left(p\left(Y_{n}\right)\right)$. Additional knowledge always reduces the attainable information production rate.

In principle, for any finite amount of data *x*(*t*) one can construct the optimal map, *π*(*n*), for the process, *X*, by minimizing over all possible sequences of parsing rules, at any fixed parsing level *n*, if only we know the relevant set of parsing rules to consider and this set is finite. Then we can in principle find *n* = n*(*t*) such that no further reduction of the minimal description length is possible by applying any more parsing rules. In practice, an extensive search over all possible sequences of parsing rules is of course not feasible, even if the set of parsing rule templates only consists of the elementary template, and algorithms for inferring adjoint representations of data need to turn to different means of optimization. For theoretical considerations we may, however, assume that for a given finite relevant set of parsing rule templates and any finite *t* we can find the optimal map *π* (or one of several possible optimal maps if the map is not unique) or at least a map reasonably close to optimal, since the number of possible maps we would have to evaluate remains finite, too. Intuitively it is clear however, given an unknown process *X* for which we cannot pre determine the respective relevant set of parsing rule templates, that one typically can no longer decide whether a map *π* is optimal or not.

However, given an optimal *π*, the adjoint i.i.d. process, *Y*, is fully characterized by its marginal distribution, *f* = *p*(*Y*), over symbols in the adjoint alphabet, $A^{*}\equiv A_{n^{*}}$, and the information production, *I*(*X*) = *k*_*Y*_*H*(*f*), is given by the Shannon entropy of *Y*. On the adjoint process space Φ* we can use the measures of Shannon entropy, cross-entropy, and Kullback-Leibler information divergence, given that we use the appropriate unit of information, *k*_*Y*_ of Eq (5). Since *Y* is i.i.d. over $A^{*}$, the adjoint space naturally belongs to the family, Φ*, of *all* i.i.d. processes over this alphabet. Further, any *Y*′ in Φ* is fully characterized by its marginal distribution, *f*′, and the pair (*f*′, *π*), determines the process *X*′ = *π*^−1^*Y*′. Hence, the process class, Φ_*π*_, that naturally generalizes a process, *X*, with adjoint i.i.d. process, *Y* = *πX*, is given by Φ_*π*_ = *π*^−1^Φ*.

This construction completes the first part of the proof that establishes that we can essentially measure information production of a process as the Shannon entropy of the adjoint process. This entropy, however uses the marginal distributions over the adjoint alphabet as arguments and can therefore not be identified directly with the generalised entropies that use the marginal distributions over the original alphabet as arguments. In the next step we will pull the information measures over the adjoint message space back to to the original message space.

### Information measures over extended alphabets

Suppose we have a process *X* with an adjoint process *Y* = *πX*. For data, *x*, and its adjoint sequence, *y* = *y*_*n*_, we obtain two histograms, *h*_*i*_(*x*), of symbols $i\in A_{0}$ and, *h*_*z*_(*y*), of symbols $z\in A_{n}$, respectively. The associated relative frequency distributions are given by *g* = *p*(*x*) = *h*(*x*)/|*x*| and *f* = *p*(*y*) = *h*(*y*)/|*y*|. Further, every symbol, *z*, represents a number of *ℓ*(*z*) symbols, *π*^−1^*z*, in the original alphabet with *π* = *π*(*n*). We define ${\bar{h}}_{i}\left(z\right)=h_{i}\left(\pi^{-1}z\right)$ as the histogram of letters $i\in A_{0}$ that are parsed together into the symbol $z\in A_{n}$. For *z* ≤ *W* where $W=|A_{0}|$, we have, ${\bar{h}}_{i}\left(z\right)=\delta_{iz}$ and $h_{i}\left(x\right)=\sum_{z\in A_{n}}{\bar{h}}_{i}\left(z\right)h_{z}\left(y\right)$ needs to hold for all $i\in A_{0}$. This provides us with constraints,

$$\begin{array}{c} h_{i}\left(x\right)=\sum_{z\in A_{n}}\;{\bar{h}}_{i}\left(z\right)h_{z}\left(y\right)\;, \end{array}$$ (6)

that we need in the next section. As a consequence, we have

$$\begin{array}{c} g_{i}\left(x\right)=\frac{\left|y\right|}{\left|x\right|}\sum_{z\in A^{*}}{\bar{h}}_{i}\left(z\right)f_{z}\left(y\right)\;. \end{array}$$ (7)

We drop the arguments (*x*) and (*y*) (or (*y*_*n*_)) from now on and distinguish histograms by their index *i* (over $A_{0}$) and *z* (over the adjoint alphabet $A^{*}=A_{n}$). Let ${\langle \ell \rangle }_{f}=\sum_{z\in A^{*}}f_{z}\ell \left(z\right)$ (note that in this notation we identify 〈*ℓ*〉_*f*_ ≡ 〈*ℓ*〉_*Y*_) and ${\langle {\bar{h}}_{i}\rangle }_{f}=\sum_{z\in A^{*}}f_{z}{\bar{h}}_{i}\left(z\right)$ be the expectation values under the distribution *f*, then, by construction, $\left|x\right|=\sum_{z\in A^{*}}h_{z}\ell \left(z\right)$, $\left|y\right|=\sum_{z\in A^{*}}h_{z}$, and $k_{Y}\left(x\right)=k{\langle \ell \rangle }_{f}^{-1}$. This means that we can write the constraints that link distributions *g* over $A_{0}$, Eq (6), with distributions *f* over $A^{*}$ in the following way

$$\begin{array}{c} 0\;=\;C_{i}\left(g|f\right)\;\equiv \;{\left\langle \ell \right\rangle }_{f}^{-1}{\left\langle {\bar{h}}_{i}\right\rangle }_{f}\;-\;g_{i}\;. \end{array}$$ (8)

As mentioned before, the process class, Φ_*π*_, that *X* belongs to, is completely determined by the map *π* and the process *X*, by the pair (*f*, *π*), see Fig 1. Therefore, we can identify the entropy of *X* with

$$\begin{array}{c} S_{\pi }\left(f\right)\equiv k_{Y}H\left(f\right)\;, \end{array}$$ (9)

with the process-specific Boltzmann factor, *k*_*Y*_ ≡ *k*/(〈*ℓ*〉_*f*_). For processes, *X* and *X*′, and with *f*′ = *p*(*πX*′), the cross-entropy and the information divergence are

$$\begin{array}{c} \begin{array}{ccc} S_{\pi }^{\text{cross}}\left(f|\right|f^{\prime }) & \equiv & k_{Y}H_{\text{cross}}\left(f|\right|f^{\prime })\\ D_{\pi }(f^{\prime }\left||f\right) & \equiv & k_{Y}D_{\text{KL}}(f^{\prime }||f)\;. \end{array} \end{array}$$ (10)

In the special case where *X* is already an i.i.d. process, no features can be extracted from the data and *n* = 0, *π* = *π*(0) = *id*, and *ℓ*(*z*) = 1, for all *z* = 1⋯*W*. Consequently, *S*_*π*_ = *kH*, $S_{\pi }^{\text{cross}}=kH_{\text{cross}}$, and $D_{\pi }=kD_{\text{KL}}$ (Kullback-Leibler divergence), as required.

### Pulling back entropies to the original alphabet

In the next step one can construct entropy functionals over the original alphabet, $A_{0}$, by lifting a distribution function, *g*′, on $A_{0}$ to a distribution function, *f*′, over $A^{*}$ by assuming that *f* = *p*(*πX*) is the true distribution function of the process, *Y* = *πX*. We proceed by minimizing the information divergence, *D*_*π*_(*f*′||*f*), with respect to *f*′. More precisely, we minimize the functional *ψ*(*f*′, *α*, *η*) given by

$$\begin{array}{c} D_{\pi }(f^{\prime }\left||f\right)-\alpha (|f^{\prime }|-1)-\sum_{i\in A_{0}}\eta_{i}\;C_{i}\left(g^{\prime }|f^{\prime }\right)\;, \end{array}$$ (11)

with Lagrange multipliers, *α* and *η*_*i*_, that normalize *f*′ and guarantee the constraints from Eq (8). Solving the variational principle *δψ* = 0 estimates *f*′ at the minimum that is compatible with *g*′. We identify this minimizer as $\hat{f}\left(g^{\prime }\right)$ and obtain *α* = 1/〈*ℓ*〉_*f*_ and

$$\begin{array}{c} {\hat{f}}_{z}\left(g^{\prime }\right)=f_{z}\;e^{(D_{\pi }(\hat{f}\left(g^{\prime }\right)\left||f\right)+\sum_{i\in A_{0}}\frac{\eta_{i}}{{\left\langle \ell \right\rangle }_{f}}(\frac{{\bar{h}}_{i}\left(z\right)}{\ell (z)}-g_{i}^{\prime }))\ell \left(z\right)}\;, \end{array}$$ (12)

which has to be solved self consistently. If *f* already meets all matching constraints with *g*′, i.e. if *g*′ = *g* with *g* = *p*(*X*), then we have $D_{\pi }(\hat{f}\left(g\right)||f)=0$ and $\hat{f}\left(g\right)=f$.

This means that one can lift marginal distributions, *g*′, on $A_{0}$ to distributions, $\hat{f}\left(g^{\prime }\right)$, on $A_{n}$ with respect to a particular process, *X*. As a consequence one can pull the entropy, cross-entropy, and divergence back from distributions, *f*, over the adjoint sample space, $A_{n}$, to distributions, *g*, over the initial alphabet, $A_{0}$, with respect to a particular process, *X* ∈ Φ_*π*_. In particular, one can define the projection operator, π_*_, through *g* = *p*(*π*^−1^*y*) ≡ π_*_p(*y*) = π_*_f and the operator, $\pi_{X}^{*}$, that lifts distributions, *g*, over alphabet, $A_{0}$, to distributions *f* over the extended alphabet, $A^{*}$, through

$$\begin{array}{c} \pi_{X}^{*}g\;\equiv \;{\text{minarg}}_{\left\{f^{\prime }\;|\;g=\pi_{*}f^{\prime }\right\}}\;D_{\pi }(f^{\prime }||p\left(\pi \;X\right))\;, \end{array}$$ (13)

with respect to the process *X*, i.e., with respect to the distribution function, *p*(*πX*), of the i.i.d. process, *πX*, over the adjoint alphabet. The lift operator gives us the minimizer $\hat{f}\left(g\right)=\pi_{X}^{*}\;g$. We find that $id=\pi_{*}\pi_{X}^{*}$ and identify

$$\begin{array}{c} \begin{array}{ccc} S_{X}\left(g\right) & \equiv & S_{\pi }\left(\pi_{X}^{*}g\right)\\ S_{X}^{\text{cross}}(g^{\prime }\left||g\right) & \equiv & S_{\pi }^{\text{cross}}(\pi_{X}^{*}g^{\prime }\left|\right|\pi_{X}^{*}g)\\ D_{X}(g^{\prime }\left||g\right) & \equiv & D_{\pi }(\pi_{X}^{*}g^{\prime }\left|\right|\pi_{X}^{*}g)\;, \end{array} \end{array}$$ (14)

where, typically, *g* = *p*(*X*). We call those measures the *pull-back* entropy, cross-entropy, and information divergence of the process *X*. Note, that while *S*_*π*_, $S_{\pi }^{\text{cross}}$, and *D*_*π*_ are universal on the entire class of processes Φ_*π*_, pulling the information measures back to marginal distributions *g* over $A_{0}$ yields information measures that are specific to a particular process *X* ∈ Φ_*π*_.

### Generalised entropies over initial alphabets

The final question is how the pull-back measures *S*_*X*_, $S_{X}^{\text{cross}}$, and *D*_*X*_, defined in Eq (14), are related to generalised entropy functionals as derived for example in [6, 21]. There, functionals were derived to obtain the most likely histogram, *h*, observed in a given process after *t* observations (for large *t*). Even for non i.i.d. processes, often the probability, *P*(*h*|*θ*), to observe the particular histogram, *h*, for *t* = ∑_*i*∈Ω_ *h*_*i*_ observations factorizes *P*(*h*|*θ*) = *M*(*h*)*G*(*h*|*θ*) into a *multiplicity*, *M*(*h*), and a probability term of the sequences, *G*(*h*|*θ*). *θ* is a set of parameters that determines the process, *X*—it defines and parametrizes the process class *X* belongs to. Whenever such a factorization is possible, one can show that a generalised maximum entropy principle exists. Using the Boltzmann definition of entropy, the logarithm of multiplicity, $\tilde{S}=\;\text{log}\;M/t$, and defining ${\tilde{S}}^{\text{cross}}=-\;\text{log}\;G/t$, and a generalised information divergence as $\tilde{D}\left(g|\theta \right)=-t^{-1}\;\text{log}\;P\left(h|\theta \right)$, where *g* = *h*/*t*, the standard relations $\tilde{D}={\tilde{S}}^{\text{cross}}-\tilde{S}$ remain valid [5].

If we are looking at a family of processes *X*(*θ*) rather than a single process *X* then we can no longer assume a priorly that the same map *π*^(1)^ that takes some process *X*(*θ*^(1)^) to an adjoint representation efficiently is the same map *π*^(2)^ that takes some process *X*(*θ*^(2)^) from the family efficiently to an adjoint representation. However, there are ways in which we can think of constructing a single map *π* to some extended alphabet $A^{*}$ such that all processes *X*(*θ*) decorrelate under the same map *π*. In the weakest version we can start with some extended alphabet $A^{*}$ and the space of all i.i.d. processes Φ* over that alphabet and a map *π* and consider the process family Φ_*π*_ = *π*^−1^Φ*, in which case the natural parameters of the processes *X*(*θ*) are given by *θ* = *f*, where *f* are distribution functions over $A^{*}$. For a discussion of stronger versions see Text 6 in S1 File.

However, if above assumption is valid, then *X*(*θ*) forms a sub-class of processes in Φ_*π*_ with distribution functions, *g*(*θ*) = *p*(*X*(*θ*)), and adjoint distribution functions, *f*(*θ*) = *p*(*πX*(*θ*)). similarly we have $\bar{\ell }\left(\theta \right)=\sum_{z}f_{z}\left(\theta \right)\ell \left(z\right)$, *y*′ = *πx*′ and $t=|x^{\prime }\left|=\right|y^{\prime }|\bar{\ell }\left(\theta \right)$. And in the next step we note that the probability of observing a histogram *h*′ under process *X*(*θ*) is given by

$$\begin{array}{lll} P(h^{\prime }|\theta ) & = & \sum_{h^{\prime }=h\left(x\right)}\;p\left(x\right)\\ & = & \sum_{h^{\prime }=h\left(\pi^{-1}y\right)}p\left(y\right)\\ & = & \sum_{h^{\prime }=\bar{h}h^{*}}\left({}_{h^{*}}^{\left|h^{*}\right|}\right)\sum_{z\in A^{*}}f_{z}{\left(\theta \right)}^{h_{z}^{*}}\\ & ≃ & {\text{max}}_{h^{\prime }=\bar{h}h^{*}}\left({}_{h^{*}}^{\frac{t}{\bar{l}\left(\theta \right)}}\right)\underset{z\in A^{*}}{\prod }f_{z}{\left(\theta \right)}^{h_{z}^{*}}\\ & = & P({\hat{h}}^{*}|f\left(\theta \right)) \end{array}$$ (15)

with ${\hat{h}}^{*}$ being the respective maximizing argument h*, the histogram over the extended space. The expression $h^{\prime }=\bar{h}h^{*}$ are the constraints between the histograms on the original and adjoint alphabet. Since *y* are sampled i.i.d, in the last line

$$\begin{array}{c} P\left(h^{*}|f\left(\theta \right)\right)=\left({}_{h^{*}}^{\frac{t}{\bar{l}\left(\theta \right)}}\right)\prod_{z\in A^{*}}\;f_{z}{\left(\theta \right)}^{h_{z}^{*}}\;. \end{array}$$ (16)

is the multinomial distribution of histograms h*. Also note that in the one but last line of Eq (15) ≃ stands for *asymptotically equivalent*, meaning, that the relative error we make in log *P*(*h*′|*θ*) by replacing *P*(*h*′|*θ*) with *P*(h*|*f*(*θ*)) vanishes in probability as *t* → ∞. For more details see Text 7 in S1 File. We note, that this asymptotic equivalence is a consequence of processes that can be mapped into an adjoint representation becoming i.i.d. there and therefore implicitly can be thought of being ergodic on sufficiently large time scales.

Taking logs and multiplying both sides with −*k*/*t* and setting *g*′ = *h*′/*t* and f* = k*/|k*|, we then obtain

$$\begin{array}{c} \begin{array}{ccc} D(g^{\prime }|\theta ) & ≃ & \min_{g^{\prime }=\bar{h}h^{*}/t}k_{Y(\theta )}D_{\text{KL}}\left(f^{*}|f\left(\theta \right)\right)\\ & = & \min_{C(g^{\prime }|f^{*})}D_{\pi }\left(f^{*}|f\left(\theta \right)\right)\\ & = & D_{X(\theta )}\left(g^{\prime }|g\left(\theta \right)\right)\;, \end{array} \end{array}$$ (17)

where ≃ again means asymptotically identical for large *t*; from the second to the third line we used the definition of the lift operator from Eq (13) and *D*_*X*_ from Eq (14). In other words, we have shown that in the limit *t* → ∞ the generalised information divergence *D*(*g*′|*θ*) is identical to the pull-back divergence *D*_*X*(*θ*)_ (*g*′|*g*(*θ*)) as a functional. As a consequence we can use that $D_{X}=S_{X}^{\text{cross}}-S_{X}$ and identify *S*_*X*_ with the generalised entropy and $S_{X}^{\text{cross}}$ with the generalised cross entropy. We see that the generalised entropy, $\tilde{S}$, for the processes family, *X*(*θ*), is given by $\tilde{S}\left(g\right)=S_{X(\theta )}\left(g\right)$.

We conclude that for all process classes (at least those that that decorrelate over a common map *π*) there exist notions of entropy, *S*_*X*_, cross-entropy, $S_{X}^{\text{cross}}$, and divergence, *D*_*X*_, that behave in the usual way, namely, $D_{X}=S_{X}^{\text{cross}}-S_{X}$. This means that for such processes (at least asymptotically) the probability to observe a particular histogram, *P* ≃ exp(−*tD*_*X*_), factorizes into a multiplicity term, *M* ≃ exp(*tS*_*X*_), associated with entropy, and a sequence probability term, $G≃\text{exp}(-tS_{X}^{\text{cross}})$, associated with cross-entropy. In other words, we have shown that Boltzmann’s entropy, the logarithm of multiplicity, remains the correct estimator of information production. For complex systems the multiplicity will differ from a multinomial coefficient in arbitrarily complicated ways that might even depend explicitly on system parameters *θ*, which would mean a violation of SK1 axiom. In other words, the Boltzmann pull-back entropy functional typically will be more complicated than the Shannon entropy functional and can even violate SK1—yet they are the appropriate generalizations of entropy, in terms of information production. We also learned that beyond such parametric families of generalised entropies, i.e. beyond the pull-back measures, we find the standard notions of entropy, cross-entropy, and divergence present in the adjoint alphabet, where the only thing that is not universal about the information measures is the process-specific Boltzmann constant, *k*_*Y*_, that needs to be used.

### Example: SSR processes

We now demonstrate explicitly that the generalised entropy that –according to the previous section– is identified with the pull-back entropy, *S*_*X*_, indeed does measure information production. We do that by considering slowly driven sample space reducing (SSR) processes, *X*, for which the generalised entropy functional is exactly known [21]. SSR processes are models of driven non-equilibrium systems. They are characterized by the fact that as the process unfolds the number of states accessible to the process reduces when no driving is present [30]. In its simplest form, the process relaxes to a ground state from which it has to be restarted. One can think of the process as a ball bouncing down a staircase with random jump sizes. The ball can only jump to steps lower than the last step it visited. Once it reaches the bottom of the staircase one lifts the ball to the top of the staircase (driving), and kicks it down the staircase again. The stairs represent (energy) states, the lowest being 1, the highest is *W*. The process exhibits path-dependence in the relaxation part, the current through the system breaks detailed balance between states. Such processes exhibit a Zipf law in their distribution function.

The micro-states, *x*, of the SSR process are sequences of states with elements *x*_*n*_ ∈ {1, 2, ⋯, *W*} ≡ Ω. The transition probabilities between states *j* → *i* are

$$\begin{array}{c} q\left(i|j\right)=\Theta \left(j-i\right)q_{i}/Q_{j-1}\;+\;\delta_{j,1}q_{i}\;\; \end{array}$$ (18)

where the first term on the right hand side describes the relaxing part of the SSR process (transitions only happen from higher *i* to lower states, *j*, i.e., when *j* < *i*) with prior distribution *q*_*i*_ and cumulative distribution, $Q_{j}=\sum_{i=1}^{j}q_{i}$. Θ is the Heavyside function. The second term captures the (slow) driving of the process. Slow here means that the system is only driven once the SSR process reaches its lowest position *i* = 1. SSR processes are Markovian since transition probabilities depend only on the current position and it is ergodic since after the relaxation process the system is reset to any state with probability, *q*_*i*_.

To understand the statistics of the process we are interested in the distribution of visits to the individual states. We define the macro-state to be the histogram, *h*_*i*_, of visits of *X* to state, *i*. It is possible to compute $S_{\text{SSR}}\left(p\right)=\frac{1}{t}\;\;\text{log}\;M\left(k\right)$, where the multiplicity, *M*(*k*), is the number of different sequences, *x*, of length *t* with the same histogram *h*. One finds [21]

$$\begin{array}{c} S_{\text{SSR}}\left(p\right)=-\sum_{i=2}^{W}\;p_{i}\;\text{log}\;\frac{p_{i}}{p_{1}}+\left(p_{1}-p_{i}\right)\;\text{log}\;\frac{p_{1}-p_{i}}{p_{1}}\;, \end{array}$$ (19)

where *p*_*i*_ = *h*_*i*_/*t* are the relative frequencies of observing a state *i*. Note that this is the Boltzmann entropy of the system, yet it is not of Shannon form since it is derived from a Markov, and not an independent sampling process. Similarly, one finds the cross-entropy

$$\begin{array}{c} S_{\text{SSR}}^{\text{cross}}\left(p|q\right)=-\sum_{i=1}^{W}\;p_{i}\;\text{log}\;q_{i}+\sum_{i=2}^{W}\;p_{i}\;\text{log}\;Q_{i-1}\;, \end{array}$$ (20)

and by maximizing $\psi =S_{\text{SSR}}-S_{\text{SSR}}^{\text{cross}}$, (negative information divergence *D*), one obtains the characteristic Zipf distribution of the slowly driven SSR process [30]

$$\begin{array}{c} p_{i}=p_{1}\frac{q_{i}}{Q_{i}}\;. \end{array}$$ (21)

For the special case of *q*_*i*_ = 1/*W* for all *i*, the Zipf distribution, $p_{i}∼\frac{1}{i}$, is obvious, since *Q*_*i*_ = *i*/*W* and *q*_*i*_/*Q*_*i*_ = 1/*i*. It continues to hold for “well-behaved” *q*_*i*_, [31]. For instance, if *q*_*i*_ ∝ *i*^*α*^ for *α* > −1, then *Q*_*i*_ ∝ *i*^1+*α*^ and again *q*_*i*_/*Q*_*i*_ ∝ 1/*i*.

In the next step we will use a simple example of a slowly driven SSR process in order to demonstrate how using extended alphabets works and how the respective generalised entropy functional is the adequate measure of information production.

#### Example of a small SSR system

To demonstrate how a minimal adjoint alphabet for a slowly driven SSR process looks like, consider such a process over an initial alphabet of *W* = 4 symbols (numbers) representing the four states, $i\in A_{0}=\left\{1,2,3,4\right\}$. A SSR sequence in that alphabet might look like *x* = 421214321431212141⋯. Remember that the *q*_*i*_ are normalized weights such that the probability to sample the state *j* < *i* conditional on the process being in state *i* is given by *q*_*j*_/*Q*_*i*_, with $Q_{i}=\sum_{j=1}^{i}q_{j}$ and by *q*_*j*_ if the system is in the ground state *i* = 1 and the system gets driven. One can think of the adjoint SSR alphabet, $A_{n^{*}}$, as the union of $A_{0}$ with the set of new symbols that represent all possible strictly monotonic decreasing sequences on $A_{0}$, i.e., $A_{n^{*}}=\left\{1,2,3,4,5,6,7,8,9,10,11\right\}$, where the new symbol “5” represents the sequence 21, “6” stands for 31, “7” for 321, “8” for 41, “9” for 421, “10” for 431, and “11” for 4321. Since we have 7 new symbols, n* = 7 extending the alphabet of original symbols {1, 2, 3, 4} we have a total of 11 symbols in the extended alphabet $A_{n^{*}}$. The 7 parsing rules producing the new symbols are given by

$$\begin{array}{c} \begin{array}{ccc} \pi_{1} & = & \left[2\;1\to 5\right]\\ \pi_{2} & = & \left[3\;1\to 6\right]\\ \pi_{3} & = & \left[3\;5\to 7\right]\\ \pi_{4} & = & \left[4\;1\to 8\right]\\ \pi_{5} & = & \left[4\;5\to 9\right]\\ \pi_{6} & = & \left[4\;6\to 10\right]\\ \pi_{7} & = & \left[4\;7\to 11\right] \end{array} \end{array}$$ (22)

and the map *π* = *π*_7_*π*_6_*π*_5_*π*_4_*π*_3_*π*_2_*π*_1_, which maps between messages written in the initial and the adjoint alphabet, can be constructed. We therefore can rewrite our example *x* = 421214321431212141⋯ into *π*(1)*x* = 4554354315541⋯, then *π*(2)*x* = 455435465541⋯, *π*(3)*x* = 45547465541⋯, *π*(4)*x* = 4554746558⋯, *π*(5)*x* = 954746558⋯, *π*(6)*x* = 9547[10]558⋯, and finally *π*(7)*x* = 95 [11][10]558⋯. We now project a distribution function, *f*, on $A_{n^{*}}$ to a distribution function, *g*, on $A_{0}$. We remember that the letters 5 to 11 code for the following subsequence: *π*^−1^5 = 21, *π*^−1^6 = 31, *π*^−1^7 = 321, *π*^−1^8 = 41, *π*^−1^9 = 421, *π*^−1^10 = 431, and *π*^−1^11 = 4321, and see that all the new letters with index 5 to 11 represent sequences-junks that contain a 1. That is ${\bar{h}}_{1}\left(z\right)=\delta_{1z}+\sum_{s=5}^{11}\delta_{sz}$. Letter 2 is part of the sequences-junks represented by the extended letters 5, 7, 9, and 11. That is ${\bar{h}}_{2}\left(z\right)=\delta_{1z}+\delta_{5z}+\delta_{7z}+\delta_{9z}+\delta_{11z}$. Similarly we can find ${\bar{h}}_{i}\left(z\right)$ for *i* = 3 and *i* = 4. As a consequence the distribution functions *g*_*i*_ of the message *x* in original letters *i* = 1, ⋯, 4 and the distribution function *f*_*z*_ of the adjoint message *π*(7)*x* in extended letters *z* = 1, 2, ⋯, 11 are given by four equations:

$$\begin{array}{c} \begin{array}{ccc} g_{1} & = & \frac{1}{Z}(f_{1}+f_{5}+f_{6}+f_{7}+\cdots \\ & & \;+f_{8}+f_{9}+f_{10}+f_{11})\\ g_{2} & = & \frac{1}{Z}\left(f_{2}+f_{5}+f_{7}+f_{9}+f_{11}\right)\\ g_{3} & = & \frac{1}{Z}\left(f_{3}+f_{6}+f_{7}+f_{10}+f_{11}\right)\\ g_{4} & = & \frac{1}{Z}\left(f_{4}+f_{8}+f_{9}+f_{10}+f_{11}\right)\;, \end{array} \end{array}$$ (23)

where *Z* is a normalization constant such that $1=\sum_{i=1}^{4}g_{i}$. Note that after applying *π* to a SSR process yields (asymptotically) that *f*_2_ = *f*_3_ = *f*_4_ = 0. We can now express the asymptotic relative frequencies, i.e. the probabilities, *f*_*z*_, in terms of the weights *q*_*i*_ on the SSR states *i* = 1, 2, 3, 4, and get, *f*_5_ = *q*_2_, *f*_6_ = *q*_3_*q*_1_/(*q*_1_ + *q*_2_), *f*_7_ = *q*_3_*q*_2_/(*q*_1_+ *q*_2_), *f*_8_ = *q*_4_*q*_1_/(*q*_1_ + *q*_2_ + *q*_3_), and so forth. Inserting the expressions for *f*_*z*_ in Eq (23) one self-consistently obtains the marginal distribution on the original alphabet

$$\begin{array}{c} g_{i}=\frac{1}{Z}\frac{q_{i}}{Q_{i}}\;, \end{array}$$ (24)

as predicted from Eq (21);–note that if *q*_*i*_ = 1/*W* is uniform, then the solution *q*_*i*_/*Q*_*i*_ = 1/*i* is exactly reproducing Zipf’s law and for a broad variety of choices for *q*_*i*_ one obtains approximate Zipf laws. That means that *f* fulfils the matching constraints of Eq (8) exactly and therefore also the lift, $\pi_{X}^{*}$, of the asymptotic marginal distribution function, *g*, to *f* is exact and is given by $f=\pi_{X}^{*}g$. That is, we can see in this simple example how the distribution function *g* over the original alphabet can be predicted from knowing the distribution function *f* of letters of the extended alphabet.

Since slowly driven SSR processes are in fact also Markov processes, meaning, they are processes where the probability to sample the the state of the process at *t* + 1 only depends on the state the system at the previous time-step *t* (compare transition probabilities Eq (18), one can also proof that the respective generalised entropies are actually the adequate information measures in this context. It is well known that the information production of a Markov process can be measured by the so-called conditional entropy, $S_{\text{cond}}$. This is a functional that depends on the probabilities *p*^(2)^ = *p*_*ij*_, that a symbol *j* follows symbol *i* in the process. The SSR entropy on the other hand depends on *p*^(1)^ = *p*_*i*_ is the marginal distribution of the symbols, *i*. If *p*^(2)^ is the maximizer of the conditional entropy, or more precisely, the minimizer of the conditional information divergence, and *p*^(1)^ is the minimizer of the SSR information divergence, then both estimators of entropy, the conditional entropy and the SSR entropy, are identical, $S_{\text{cond}}\left(p^{\left(2\right)}\right)\equiv S_{\text{SSR}}\left(p^{\left(1\right)}\right)$, for all choices of the system parameters *q*. For details of the computation, see Text 5 in S1 File.

## Discussion

We showed that by identifying the entropy of a process with its information production it is possible to consistently extend the fundamental notion of entropy in statistical physics –Boltzmann entropy– to non-i.i.d. processes and processes that operate out of equilibrium. This is done by identifying isomorphisms that map entire process classes to adjoint representations where processes are i.i.d. The sample space (or alphabet) of the adjoint process is typically much larger than the sample space of the original process. The isomorphisms can be thought of concatenations of parsing rules that map strongly correlated segments in the original process to new symbols. Information production of the adjoint i.i.d. process is quantified by Shannon entropy. Pulling back the entropy measure in the adjoint space to the original sample space and comparing the resulting functional with the Boltzmann entropy (process-specific log multiplicity) establishes the asymptotic equivalence of the notion of generalised entropy and information production.

This provides a comprehensive image that consistently links information theory and the statistical physics of categorial non i.i.d. processes in a context-sensitive way that allows us to consistently associate a notion of entropy, a cross-entropy (representing the constraints of the maximum entropy principle), and an information divergence (or relative entropy) to complex processes. Context-sensitive means that the functional form of the entropy depends on the class of processes considered; the concept of entropy itself, information production from the information theoretic perspective and Boltzmann entropy from the physics perspective, remains untouched.

If an adjoint representation of one process is found, one can find the adjoint representations of an entire class of processes that all de-correlate in their representations over the same adjoint sample space. This means that there exists a natural way how processes implicitly define their own generalization to an entire process class. This is possible because the property of de-correlating over the same adjoint sample space implements an equivalence relation. This has important consequences since these equivalence classes of processes generalize the idea of an ensemble to non-i.i.d. processes which provides a concise way –grounded in first principles– to extend the program of statistical physics to complex processes and their macro variables.

## Supporting information

S1 File The supporting informations supply seven texts that cover: SI Text 1 Shannon’s example of random texts from different alphabets: Here Shannon’s examples from his seminal paper about information theory are given in order to give an intuitive demonstration on the effects of extending alphabets e.g. from letters to words. SI Text 2 Minimal description length, i.i.d. processes, and compression A brief discussion on how compression works for i.i.d. processes. SI Text 3 Generative grammars, parsing rules and parsing rule templates: A brief discussion of the role parsing rules and their role in Generative grammars. We also discuss more broadly how we distinguish a parsing rule template from a particular parsing rule. SI Text 4 Information production and Kolmogorov complexity: Some remarks on how Information production relates to Kolmogorov complexity. [SI Text 5 Detailed algebraic steps for Eq. (29): The algebraic steps leading up to Eq (29) of this paper are given in detail. SI Text 6 About conjugate representations of process families: A brief discussion of issues concerning the existence of adjoint representation of entire process families rather than adjoint representations of a single process. SI Text 7 Measure concentration, typicality and asymptotic equivalence: A more detailed discussion of what asymptotic equivalence means in Eq. (15), explaining in which sense the generalized information measures based on Boltzmann entropy are equivalent to the pull-back information measures derived in this paper.
(PDF)
