# Supplementary material for: Equivalence of information production and generalised entropies in complex processes
Source: PLoS One. 2023 Sep 6;18(9):e0290695. doi: 10.1371/journal.pone.0290695 (PMC10482297; doi:10.1371/journal.pone.0290695)
Supplement: S1 File — The supporting informations supply seven texts that cover: SI Text 1 Shannon’s example of random texts from different alphabets: Here Shannon’s examples from his seminal paper about information theory are given in order to give an intuitive demonstration on the effects of extending alphabets e.g. from letters to words. SI Text 2 Minimal description length, i.i.d. processes, and compression A brief discussion on how compression works for i.i.d. processes. SI Text 3 Generative grammars, parsing rules and parsing rule templates: A brief discussion of the role parsing rules and their role in Generative grammars. We also discuss more broadly how we distinguish a parsing rule template from a particular parsing rule. SI Text 4 Information production and Kolmogorov complexity: Some remarks on how Information production relates to Kolmogorov complexity. [SI Text 5 Detailed algebraic steps for Eq. (29): The algebraic steps leading up to Eq (29) of this paper are given in detail. SI Text 6 About conjugate representations of process families: A brief discussion of issues concerning the existence of adjoint representation of entire process families rather than adjoint representations of a single process. SI Text 7 Measure concentration, typicality and asymptotic equivalence: A more detailed discussion of what asymptotic equivalence means in Eq. (15), explaining in which sense the generalized information measures based on Boltzmann entropy are equivalent to the pull-back information measures derived in this paper. (PDF) [file pone.0290695.s001.pdf]

Supplementing Information

SI Text 1: Shannon's example of random texts from different alphabets

Shannon develops his intuition underlying his definition of information production from examples he takes from language, i.e. how differently artificially generated text looks like, if one samples text using the English alphabet $\mathcal{A}_{\mathrm{letter}}$ and (1) only uses the marginal distribution $p(x_t)$, with $a = x_t$ being letters $a \in \mathcal{A}_{\mathrm{letter}}$, here is the example Shannon gives:

"OCRO HLI RGWR NMIELWIS EU LL NBNESEBYA TH EEI ALHENHTTPA OOBTTVA NAH BRL",

or if (2) one uses information on letter transition probabilities $p(a'|a)$, for letter $a, a' \in \mathcal{A}_0$ in English texts,

"ON IE ANTSOUTINYS ARE T INCTORE ST BE S DEAMY ACHIN D ILONASIVE TUCOOWE AT TEASONARE FUSO TIZIN ANDY TOBE SEACE CTISBE",

or if one (3) switches to the word level with the alphabet $\mathcal{A}_{\mathrm{word}}$ of English words and samples $p(x_t)$, with $w = x_t$ being words $w \in \mathcal{A}_{\mathrm{word}}$,

"REPRESENTING AND SPEEDILY IS AN GOOD APT OR COME CAN DIFFERENT NATURAL HERE HE THE A IN CAME THE TOOF TO EXPERT GRAY COME TO FURNISHES THE LINE MESSAGE HAD BE THESE",

or if (4) one uses information on letter transition probabilities $p(w'|w)$, for words $w, w' \in \mathcal{A}_{\mathrm{word}}$ in English texts,

"THE HEAD AND IN FRONTAL ATTACK ON AN ENGLISH WRITER THAT THE CHARACTER OF THIS POINT IS THEREFORE ANOTHER METHOD FOR THE LETTERS THAT THE TIME OF WHO EVER TOLD THE PROBLEM FOR AN UNEXPECTED".

SI Text 2: Minimal description length, i.i.d. processes, and compression

Let us briefly look at how embedding messages into larger alphabets works for compressing i.i.d. sequences. The Kraft-McMillan theorem tells us that we can find a uniquely decodable prefix code over a code alphabet of length r (typically binary $r = 2$) for the states $i \in \Omega = \{1, \cdots, W\}$ with length of the codewords ℓ_i if and only if $\sum_{i=1}^{W} r^{-\ell_i}$. Let $h_i(x)$ be the number of times the symbol i appears in the message x and $p_i = h_i/t$ is the marginal frequency distribution of states i in the same message. If one chooses $\ell_i = \lceil \log(1/p_i)/\log r \rceil$, where $\lceil \log(1/p_i) \rceil$ is the natural number such that $\lceil y \rceil \;\geq\; y \;>\; \lceil y \rceil - 1$, then those ℓ_i satisfy the Kraft-McMillan theorem and for the description length $L(x)$ of the process we get that $L = \sum_{i=1}^{W} h_i \lceil \log(1/p_i)/\log r \rceil$ and as a consequence we obtain

$$H(p)/\log r + 1 > \frac{L}{t} \geq H(p)/\log r \qquad (1)$$

where $H(p) = \sum_{i=1}^{W} p_i \log(1/p_i)$ is Shannon entropy. Since we assume the process is i.i.d. then $H(p)/\log r$ is also the minimal description length (MDL) per symbol $i = 1, \cdots, W$ that can be asymptotically achieved as the data volume, $t = |x|$, gets large.

However, MDL typically cannot be fully obtained for a message written in its original alphabet. A binary process emitting only zeros and ones cannot be made any shorter by encoding single zeros and ones differently. The information theoretic way to show that $H(p)/\log r$ is the asymptotically obtainable lower limit of the MDL, is by considering extended alphabets, for instance Ω^2 with new letters $i' = ij \in \Omega^2$ that are 2-tuples of the original letters. If the process is i.i.d. then we also know that for instance the probability of $p'_{i'} = p_i p_j$ and from the additivity of H we obtain $H(p') = 2H(p)$. If we transform the message of even length t, $x(t) = x_t x_{t-1} x_{t-2} \cdots x_1$, into a sequence of half the length written in 2-tuple letters $x'(t/2) = (x_t x_{t-1})(x_{t-2} x_{t-3}) \cdots (x_2 x_1)$, we have $t \to t' = t/2$ and we get that $H(p')/\log r + 1 > \frac{L}{t'} \geq H(p)/\log r$. As a consequence we get for the MDL L, in the 2-tuple alphabet Ω^2, that $H(p)/\log r + 1/2 > \frac{L}{t} \geq H(p)/\log r$. Considering 2^n-tuple letters $i' \in \Omega^{(2^n)}$ one gets for the effective minimal information rate that $H(p)/\log r + 2^{-n} > \frac{L}{t} \geq H(p)/\log r$. In other words, by considering larger and larger alphabets one can rewrite i.i.d. messages into a code alphabet (something we will never do here) in such a way that asymptotically one finds that $\frac{L}{t} \simeq H(p)/\log r$, asymptotically approaching the lower bound from above. The same asymptotic result can be obtained for i.i.d. processes by using parsing maps $\pi(n) = \pi_n \pi_{n-1} \cdots \pi_1$, similar to the way described in the main paper, only that we search for parses that reduce the description length without the requirement that the reduction in description length is higher then the one expected for a respective i.i.d. process. however, in this way parsing is used for pure data compression since i.i.d. data has no inherent features.

SI Text 3: Generative grammars, parsing rules and parsing rule templates

Intuitively parsing rules are particular rules that tell us how to replace some symbols in a sequence, signal, or text, with other symbols. A parsing rule template characterizes not the particular rule but the way substitution rules are constructed.

Say for instance, you look for occurrences of the letter b, that we indicate as symbol (b), following the letter a, i.e. the symbol (a), in an English text body, then glue them together to form a new symbol (ab) and replace occurrences of symbols $(a)(b)$ in your text body with (ab). In this case you add the symbol (ab) to your alphabet. The parsing depth of the alphabet rises from n to $n + 1$. If in this extended alphabet you already find another symbol $(solute)$, then in order to get the next larger alphabet you could apply the substitution $(ab)(solute)$ to $(absolute)$. The particular substitution transformation $(a)(b) \to (ab)$ implements a parsing rule, the structural shape of the parsing rule $[i\ j \to k]$, with i, j, and k being variables for symbols, implements a parsing rule template. We will call $[i\ j \to k]$ the elementary template. In fact one has to also specify how indices get selected to become unique and invertible. For a parsing rule to be invertible we want to be able to perform the inverse substitution rule$[k \to i\ j] = [i\ j \to k]^{-1}$, i.e. to expand symbol k into the subsequent occurrence of letters ij. For this to be possible only one parsing rule that produces a particular symbol k may exist and i and j already have to exist to be selected. So if we identify i, j, and k as the index of the letter, and we have already have indices $1 \cdots W$, then we can choose any $i, j \leq W$ and identify $k = W + 1$. In this case we extended the base alphabet $\mathcal{A}_0$ containing W symbols to $\mathcal{A}_1$ containing $W + 1$ symbols. In general we extend $\mathcal{A}_n$ containing $W + n$ symbols to $\mathcal{A}_{n+1}$ containing $W + n + 1$ symbols.

Parsing rules and templates: an example: Consider again English text all written solely in lower case letters so that our original alphabet consists of $W = 27$ symbols, $1 \equiv (a)$, $2 \equiv (b)$, $\cdots$, $26 \equiv (z), 27 = \text{SPACE}$. We therefore start with $\mathcal{A}_n$ with $n = 0$. One might find the following sequence of parsing rules $[(w)(o) \to (wo)]$,

$[(wo)(r) \rightarrow (wor)]$ and $[(wor)(d) \rightarrow (word)]$ in our sequence of parsing rules linking the letter to the word level alphabet. Those rules would rewrite all subsequent occurrences of the letter w, o, r, and d in an English text body written in Latin letters with the symbol $(word)$ in the word level alphabet. The parsing rules we show here follow, what we call the *elementary* parsing rule template $[i\ j \rightarrow k]$, with $i \equiv (\lambda_1)$ and $j \equiv (\lambda_2)$, and $k \equiv (\lambda_1\lambda_2)$ being variables for symbols. In this words Lempel-Ziv codecs [1] essentially rely on the elementary parsing rule template, however, with the aim to compress (see above) and not primarily for efficiently extracting features. Taking the first parsing rule $[(w)(o) \rightarrow (wo)]$ that extends the original alphabet identifies the new symbol $28 = W + n + 1 \equiv (wo)$ and the parsing rule taken from the template $[i\ j \rightarrow k]$ reads $[15\ 23 \rightarrow 28]$, where $15 \equiv (o)$ and $23 \equiv (w)$. 28 is the new symbol index in the extended alphabet $n = 1$. Every new parsing on an alphabet $\mathcal{A}_n$ will yield a new symbol with index $k = W + n + 1$.

More general parsing rules: However, in principle, depending on the process in question, one might also need to consider arbitrary complex templates. For instance, to capture clauses of the form: " **if** this **then** that", where "this" and "that" represents a text of variable length. The associated parsing rule template could read $i\ X\ j\ \rightarrow\ k[X]$ such that we can reversibly parse "(**if**)(this)(**then**)(that) $\rightarrow$ (**if then**)[(this)](that)". Note that we require additional parenthesis symbols [and] in order to make the parsing rule reversible.

Note also that we use parsing rules in the "analytic mode", $[i\ j \rightarrow k]$, when we extend an alphabet by one symbol. We use a parsing rule in the "generative mode" $[k \rightarrow i\ j]$ if we expand symbols $k = W + n + 1$ to map messages over $\mathcal{A}_{n+1}$ to messages over $\mathcal{A}_n$.

Parsing rules have been studied intensively in the field of theoretical Linguistics as methods of generating text in terms of what are called generative grammars, [2]. To give a simple example: the parsing rules $X \rightarrow aX$ and $X \rightarrow bX$, with terminal $X \rightarrow \epsilon$, where ϵ is the *empty symbol*, can be used to write any sequence of a's and b's; e.g.: $X \rightarrow aX \rightarrow aaX \rightarrow aabX \rightarrow \cdots \rightarrow aababbbaabaX \rightarrow aababbbaaba$. The two rules $X \rightarrow aX$ and $X \rightarrow Xb$ with the same terminal rule, on the other hand, would only produce sequences of the form $a^m b^n$, i.e. $X \rightarrow aX \rightarrow aaX \rightarrow aaXb \rightarrow \cdots \rightarrow aaaaaaXbbbb \rightarrow aaaaaabbbb$. It is a major achievement in this line of theoretical work that generative grammars can be classified in four hierarchically inclusive classes, [2, 3], of grammars being so called regular grammars, and at the top level one finds everything you could write as a computer program on a universal touring machine with unbounded memory (recursively enumerable languages).

SI Text 4: Information production and Kolmogorov complexity

Kolmogorov complexity and information production are two closely related concepts. The one, Kolmogorov complexity, [4–6], basically refers to the length of the shortest program (in some universal computing language, that in fact can be though of to be generated by a particular generative grammar and its associated parsing rules set to generative mode. Think for instance, the program of a standard random number generator y as it is implemented in many computing Languages, or as they can be found in *Numerical recipes in C. The art of scientific computing* (Press, William H et al, Cambridge University Press 1986, 1992). Those codes are relatively short, i.e. their Kolmogorov complexity is finite, and if run, produce pseudo random numbers. The entropy of the pseudo random number sequence, being deterministic and periodic with an extremely long period, is actually vanishing, if we had sufficient data to infer the periodicity of the signal. However, those numbers in general are astronomically large and although theoretically one could observe the periodic structure, practically this is not possible, i.e. the deterministic numbers of sequences x produced by y can hardly be

distinguished from actual random number sequences by statistical test. So if you know the generator y and the current random seed s you can perfectly predict the next number the generator emits and how the seed s updates, i.e. you can predict (y, s) translated into a number $x(t)$ the generator emits at the t'h step. That is, in order to find the true information production of the process, we would have to reconstruct parsing rules that in the end could transform data back into the code of the random number generator and its initial random seed in order to do so. In this case data x, in the infinite size limit would always be transformed back into a finite length $L(Y)$, which essentially corresponds to the code of the random number generator $Y \equiv y$ characterized by the pair (y, s), y being the code and s the seed value. That is, we understand $X = \pi Y$ to be the process we observe, which emits the data x, and π describes the hardware that translates Y into X. As a consequence, the information production, up to possibly a constant, is given by $I(X) = \lim_{t\to\infty} L(Y)/t = 0$. In other words, if we have a consistent method to extract structure from data, such that asymptotically $I(x) \to I(X)$, then the Kolmogorov complexity $L(y) \sim tI(x)$ is essentially the minimal description length of the data. In general we can expect that asymptotically $L(y) \propto t^\alpha$ for some exponent $0 \leq \alpha \leq 1$, measuring "how deterministic a process is", $\alpha = 0$ being deterministic programs (including pseudo random number generators) and $\alpha = 1$, random processes with a finite information production.

It is however more than doubtful that it is possible to reconstruct the parsing rules of arbitrary complex generative grammars purely from statistical analysis of the data they generate alone. This issue touches Chaitin's incompleteness theorem, [4], which essentially states that above a certain string complexity it is no longer possible to decide whether a string is complex or not, i.e. whether it is still compressible or not. Intuitively we would assume that for our random generator example it is probably impossible to reconstruct some version of the random number generator program from the pseydo random numbers it generates, at least if you do not a priorly know that those numbers have been produced by a random number generator.

SI Text 5: Detailed algebraic steps for Eq. (5)

Since we are dealing with a Markov process we can compute the information production also through the conditional entropy, that as we will see, leads to the same maximum configuration of marginal distributions of state visits. We focus on the joint probabilities $p(i, j)$ to observe i following j. From $p(i, j)$ one gets the marginal distribution, p_i, by marginalization. The question is how many sequences x exist when we observe a joint histogram $h(i, j) = Np(i, j)$. In other words, what is the multiplicity, M, of possible sequences x and its associated reduced Boltzmann entropy S and cross entropy $S^{\rm cross}$ if we the underlying Markov process is characterized by the transition probabilities, $q(i|j)$. It is not difficult to see that $S = \log(M)/N$, is given by the conditional entropy

$$S_{\rm cond}[p] \;=\; -\sum_{i,j=1}^{W} p(i,j)\;\log\frac{p(i,j)}{\sum_{m=1}^{W} p(m,j)}\,. \qquad (2)$$

Similarly, the cross entropy of the Markov process is found

$$S^{\rm cross}_{\rm cond}(p|q) \;=\; -\sum_{i,j=1}^{W} p(i,j)\,\log q(i|j)\,. \qquad (3)$$

Note, that $p(i, j)$ is the joint frequency distribution of transitions $j \to i$ in a sequence x, and $q(i|j)$ is the conditional probability distribution defining the Markov process. Maximizing $S_{\rm cond} - S^{\rm cross}_{\rm cond}$ (the negative conditional information divergence) with

respect to the joint distribution p under the constraint, $\sum_{i,j} p(i,j) = 1$, yields the expected result for the maximiser

$$p(i|j) \;=\; q(i|j)\,, \tag{4}$$

and the transition probabilities $q(i|j)$ of the Markov process can be estimated asymptotically (large N) by the observed empirical conditional probabilities $p(i,j)/p_j$. If we do this for the slowly driven SSR process, then $p(i|j) = q(i|j)$ and the maximizing marginal distribution p_i is obtained by solving the eigenvector equation $p_i = \sum_j q(i|j)p_j$. Since the conditional entropy, $S_{\rm cond}$, from Eq. (2) is the log of the multiplicity of sequences compatible with the empirical joint distribution $p(i,j)$, its value should equal the value of $S_{\rm SSR}$ for the marginal distribution p_i from Eq. (**??**), which is the corresponding maximizer. Denoting the maximizer of the joint distribution by $p^{(2)}$ and the one of the marginal distribution by $p^{(1)}$, and using $\frac{q_j}{Q_{j-1}Q_j} \;=\; \frac{1}{Q_{j-1}} - \frac{1}{Q_j}$, within a few algebraic steps (see below) we see that indeed

$$S_{\rm SSR}(p^{(1)}) \;=\; S_{\rm cond}(p^{(2)}) \tag{5}$$

holds identically for $q(i|j)$ from Eq. (**??**), for all choices of weights $q_j \;\geq\; 0$ and $\sum_i q_i \;=\; 1$. This implies that entropy in the context of complex processes can be approached exactly by means of *information theory* and that the existence of generalised entropies is a consequence of the complex, non-i.i.d. structure of the underlying systems and processes.

Few algebraic steps: We first of all note that in maximum configuration the marginal distribution of the SSR process is given by $p_i^{(1)} = p_1^{(1)} q_i/Q_i$, where $p_1^{(1)} = 1/Z$ acts as a normalization constant, q_i is the weight distribution and $Q_i = \sum_{j=1}^{i} q_j$ is the cumulative weight distribution of the SSR process; $i = 1, 2, \cdots, W$.

Using $p^2(i,j) = q(i|j)p_j^{(1)}$ and Eq. (**??**), we can compute

$$\begin{array}{lcl} S_{\rm cond}(p^{(2)}) & = & -\sum_{i,j=1}^{W} p_i^{(1)} q(i|j)\ \log\left(q(i|j)\right) \\ & = & (A+B+C)/Z\,, \\ A & = & -\sum_{i<j} \frac{q_j}{Q_j}\frac{q_i}{Q_{j-1}}\ \log\left(q_i\right)\,, \\ B & = & \sum_{i<j} \frac{q_j}{Q_j}\frac{q_i}{Q_{j-1}}\ \log\left(Q_{j-1}\right)\,, \\ C & = & H(q)\,. \end{array} \tag{6}$$

For term A we can use that

$$\frac{q_i}{Q_i Q_{i-1}} = \frac{1}{Q_{i-1}} - \frac{1}{Q_i} \tag{7}$$

and $Q_W = 1$ to compute A to be given by

$$\begin{array}{l} \sum_{i=1}^{W-1}\sum_{j=i+1}^{W}\left(\frac{1}{Q_{j-1}} - \frac{1}{Q_j}\right) q_i \log q_i = \\ = \sum_{i=1}^{W-1}\left(\frac{1}{Q_i} - 1\right) q_i \log q_i \\ = \sum_{i=1}^{W-1}\frac{1}{Q_i} q_i \log q_i \;-\; H(q)\,, \end{array} \tag{8}$$

Similarly, we get B;

$$\begin{array}{l} \sum_{i=1}^{W-1}\sum_{j=i+1}^{W}\left(\frac{\log Q_{j-1}}{Q_{j-1}} - \frac{\log Q_{j-1}}{Q_j}\right) q_i = \\ = \sum_{i=1}^{W-1} q_i \left(\frac{\log Q_i}{Q_i} - \sum_{j=i+1}^{W}\frac{1}{Q_j}\log\left(1 - \frac{q_j}{Q_j}\right)\right) \\ = \sum_{i=1}^{W-1} q_i \frac{\log Q_i}{Q_i} - \sum_{i=2}^{W}\left(1 - \frac{q_i}{Q_i}\right)\log\left(1 - \frac{q_i}{Q_i}\right) \end{array} \tag{9}$$

Inserting the terms A, B, and C into Eq. (6) one gets

$$\begin{array}{rcl} Z\ S_{\rm cond}(p^{(2)}) & = & -\sum_{i=1}^{W-1}\frac{q_i}{Q_i}\log\left(\frac{q_i}{Q_i}\right) \\ & & -\sum_{i=2}^{W}\left(1-\frac{q_i}{Q_i}\right)\log\left(1-\frac{q_i}{Q_i}\right) \\ & & -q_W\log(q_W)\ . \end{array} \tag{10}$$

Again, $p_i^{(1)}=p_1^{(1)}q_i/Q_i$ and $p_1^{(1)}=1/Z$. Also $Q_W=1$ and $q_1=Q_1$. One obtains that $S_{\rm cond}(p^{(2)})$ equals to

$$-\sum_{i=2}^{W}\left[p_i^{(1)}\log\frac{p_i^{(1)}}{p_1^{(1)}}+(p_1^{(1)}-p_i^{(1)})\log\left(1-\frac{p_i^{(1)}}{p_1^{(1)}}\right)\right]\ . \tag{11}$$

This however is exactly $S_{\rm SSR}(p^{(1)})$ and therefore it follows that $S_{\rm cond}(p^{(2)})=S_{\rm SSR}(p^{(1)})$ for all possible choices of weights q.

SI Text 6: About conjugate representations of process families

We noted, that by looking at a family of processes $X(\theta)$ rather than a single process X we can no longer a priorly assume that the a map $\pi^{(1)}$ that takes some process $X(\theta^{(1)})$ from the family to an adjoint representation with a (close to) minimal alphabet will be the same map $\pi^{(2)}$ that takes some process $X(\theta^{(2)})$ from the family efficiently to an adjoint representation.

We noted that we can, of course, find such self-consistent process families by already starting with some adjoint extended alphabet $\mathcal{A}^*$ with the space of all i.i.d. processes Φ^* over this alphabet and some map π and consider the process family $X(\theta)$ spanned by $\Phi_\pi=\pi^{-1}\Phi^*$, which has the natural parametrization $\theta=f$, where f are distribution functions over $\mathcal{A}^*$. We of course can also consider sub-familes $f=f(\theta)$ thereof.

The question is, can we do better, i.e. can we, in principle, find adjoint representations of entire process families $X(\theta)$ as we would find an adjoint representation for a particular process X, at least hypothetically. The problem here, we believe, is akin to training an encoder on different ensembles of data. Do we look at only Shakespeare plays or all plays by English playwrights. The map π then no longer can be the Shakespeare map $\pi_{\rm Shakespeare}$ or the $\pi_{\rm BernardShaw}$ map but has to be the map $\pi_{EnglishPlayWrights}$, which we may conveniently consider as a process of its own with Shakespeare or Bernard Shaw being two possible realisations of the same process rather than being two distinct processes. However, once we have a viable map $\pi_{\rm EnglishPlayWrights}$ and $f_{EnglishPlayWrights}$, then we can characterise Shakespeare by $f_{\rm Shakespeare}$ and Bernard Shaw by $f_{\rm BernardShaw}$. Following this intuition, one could, at least if the parameter space of θ is bounded, construct sequences of sum-processes $X^{(n)}$ such that we sample junks of length $t(n)$ (where $t(n)\to\infty$ as $n\to\infty$) for n parameter samples θ_n that were drawn evenly from the parameter space and concatenate those samples into a single process $X^{(n)}$. One should note that such a procedure would make the processes $X^{(n)}$ ergodic on a time scale $t(n)$, i.e. we translate the given ensemble into ergodic processes As we make n large we would start to sample the parameter space densely and we asymptotically obtain a map π for the entire process family $X(\theta)$.

There are of course many factors (including time and memory) that limit this method in terms of practical application. We also would suspect that for many process classes $X(\theta)$, the elementary parsing rule template no longer forms a relevant set of templates and we will in general require context sensitive parsing rule templates to construct adjoint representations and extract information efficiently. The basic idea here would be to construct a map $\pi_{\rm BernardShaw}$ and $\pi_{\rm Shakespeare}$ and so on, then interleave the extended alphabets together with the respective parsing rules that constitute the various maps $\pi_{\rm Author}$ and make them context sensitive in the sense that a

rule gets executed at some places of some sum process $X^{(n)}$ where it applies while not at others, depending on local context, such that in a Shakespearean context also Shakespearean parsing rules are favoured while in a Bernard Shaw like context Bernard Shaw parsing rules get favoured.

To which extent such a program can be automated to extract context sensitive parsing rules on statistical grounds from data is of course an open question that touch decidability issues that are known to fundamentally limit the mathematical possibility to decide whether what we see in a data stream is noise or a signal emitted by a complicated source. This may also be reminiscent of so called no-free-lunch theorems which in principle state that on the set of all possible (utility) landscape any search algorithm performs equally good or bad (however one wants to see this). So we have to know something about the structure of the processes for which we want to construct adjoint representations in order to efficiently do so. In other words, if I give you a sequence of numbers generated by a random number generator on a computer but do not tell you how I obtained those numbers you will, after a few tests conclude that the data is noise. However if I tell you that the numbers have been produced by a deterministic random number generator with double precision you could in principle sample the numbers until they start to repeat and then reconstruct with some trial and error a random number generator and a random seed that reproduces the given sequence exactly.

SI Text 7: Measure concentration, typicality and asymptotic equivalence

In the main text of the paper we compare generalized divergence from earlier work deriving from the probability, $P(h|\theta)$, of observing a histogram h_i of letters i emitted by some process $X(\theta)$ of a process class in Φ_π with the pull-back divergence, we derived in this work via adjoint representations of a process, with distribution functions, $g(\theta) = p(X(\theta))$, and adjoint distribution functions, $f(\theta) = p(\pi X(\theta))$; also $\bar{\ell}(\theta) = \sum_z f_z(\theta)\ell(z)$, $y' = \pi x'$ and $t = |x'| = |y'|\bar{\ell}(\theta)$. We computed that the probability of observing a histogram h' under process $X(\theta)$ is given by

$$\begin{array}{rcl} P(h'|\theta) & = & \sum_{h'=h(x)} p(x) \\ & = & \sum_{h'=h(\pi^{-1}y)} p(y) \\ & = & \sum_{h'=\bar{h}h^*} \binom{|h^*|}{h^*} prod_{z\in\mathcal{A}^*} f_z(\theta)^{h^*_z} \\ & \simeq & \max_{h'=\bar{h}h^*} \binom{\frac{t}{\bar{\ell}(\theta)}}{h^*} \prod_{z\in\mathcal{A}^*} f_z(\theta)^{h^*_z} \\ & = & P(\hat{h}^*|f(\theta)) \end{array} \qquad (12)$$

with $\hat{h}^*$ being the respective maximizing argument h^*. Here we clarify the $\simeq$ (asymptotic equivalence) we invoke in line 4. Using that h^* is multinomially distributed:

$$P(h^*|f(\theta)) = \binom{\frac{t}{\bar{\ell}(\theta)}}{h^*} \prod_{z\in\mathcal{A}^*} f_z(\theta)^{h^*_z} \,. \qquad (13)$$

We can write

$$-\frac{k}{t}\log P(h'|\theta) = -\frac{k}{t}\log P(\hat{h}^*|f(\theta)) - \frac{k}{t}\log\left(\frac{P(h'|\theta)}{P(\hat{h}^*|f(\theta))}\right) \qquad (14)$$

and claim for assymptotic equivalence that

$$\lim_{t\to\infty}\frac{k}{t}\log\left(\frac{P(h'|\theta)}{P(\hat{h}^*|f(\theta))}\right) = 0\,. \qquad (15)$$

An exact proof tends to become longish, but the general idea is to note that for large t the multinomial can be represented by a Gaussian around the maximum and therefore the sum over the multinomial contributions that constitute $P(h'|\theta)$ can be bounded from above by a term $P(\hat{h}^*|f(\theta)V$, where V is a sufficiently large volume around the maximum which typically can be chosen to be proportional to $t^{W^*\alpha}$ with $1/2 < \alpha < 1$, which implies that

$$\frac{k}{t}\log\left(\frac{P(h'|\theta)}{P(\hat{h}^*|f(\theta))}\right) \propto W^* \log t/t\,. \tag{16}$$

This means that if the size of the adjoint alphabet, W^*, grows with the amount of data t weaker than $t/\log t$, which is true for many processes (if we think of Heaps' law), this term vanishes as we intended to show.

References

1. Ziv J and Lempel A 1978 Compression of individual sequences via variable-rate coding *IEEE Trans. Info. Theory* **24** 530–536.

2. Chomsky N 1956 Three models for the description of language *IRE Trans. Info. Theory* **2** 113–124.

3. Chomsky N and Schützenberger M P 1963 *The algebraic theory of context free languages*, in Braffort P, Hirschberg D (eds.) Computer Programming and Formal Systems. Amsterdam: North Holland. 118–161.

4. Chaitin G J 1974 Information-theoretic limitations of formal systems *J. of the ACM* **21** 403–434.

5. Kolmogorov A N 1963 On Tables of Random Numbers. reprint in *Sankhya: The Indian Journal of Statistics* A **25** 369–376.
   Kolmogorov A N 1965 Three Approaches to the Quantitative Definition of Information *Problems Inform. Trans.*. **1** 1–7.

6. Solomonoff R. 1964. A Formal Theory of Inductive Inference Part I & II *Information and Control* **7** 1–22 & 224–254.
